# Effects of a randomized controlled hiking intervention on daily activities, sleep, and stress among adults during the COVID-19 pandemic

**DOI:** 10.1186/s12889-023-15696-7

**Published:** 2023-05-15

**Authors:** Stephanie Anzman-Frasca, Julia Drozdowsky, Callista Zayatz, Katherine Holmbeck

**Affiliations:** 1 Department of Pediatrics, Jacobs School of Medicine and Biomedical Sciences, University at Buffalo, Buffalo, NY USA; 2 Center for Ingestive Behavior Research, University at Buffalo, Buffalo, NY USA; 3 Neuroscience Program, Bucknell University, Lewisburg, PA USA

**Keywords:** Physical activity, COVID-19, Stress, Sleep

## Abstract

**Background:**

Physical activity promotes health, and physical activity done outdoors in nature may be particularly beneficial. We conducted two randomized studies to examine the implementation of a winter hiking intervention and whether this intervention affected activity choices and aspects of well-being during the COVID-19 pandemic.

**Methods:**

Convenience samples of adults (n = 53; n = 51) were recruited into two different randomized studies in 2021 and 2022 respectively. Participants completed online surveys at baseline and 6 and 11–12 weeks later. Participants were randomized to a study group (intervention or control) shortly after the baseline assessments. In both studies, the intervention group received free access to a regional winter hiking challenge. In the second study, we also provided winter traction cleats to this group to facilitate engagement in the hiking challenge. Descriptive statistics were used to summarize intervention implementation, including participants’ engagement in challenge hikes. Repeated measures ANOVA models were used to test intervention effects on key outcome variables, including hiking frequency via the Pleasant Activities List, stress via the Perceived Stress Scale, and sleep duration via the Pittsburgh Sleep Quality Index.

**Results:**

In the first study, the intervention group’s engagement in challenge hikes was low (38.5%); reported barriers included access to winter hiking equipment. In the second study, when winter traction cleats were provided, engagement in the intervention was higher, and the intervention increased hiking frequency and improved sleep. There were no significant intervention effects on stress, but the direction of effects was in the expected direction.

**Conclusions:**

Results highlight some potential positive impacts of this intervention designed to facilitate access to winter hiking. Future research could examine whether effects are stronger in a larger sample, in which additional barriers to engagement are addressed.

**Trial registration:**

This study was registered at clinicaltrials.gov on 28/12/2020 prior to participant enrollment (NCT04685681), https://clinicaltrials.gov/ct2/show/NCT04685681.

**Supplementary Information:**

The online version contains supplementary material available at 10.1186/s12889-023-15696-7.

## Introduction

Physical activity promotes positive physical and mental health outcomes, with evidence that outdoor activity may be particularly beneficial [1–4]. The COVID-19 pandemic brought many health challenges aside from the direct impacts of the virus, including decreased physical activity, increased stress, decreased sleep duration and quality, and disruptions to daily routines [5–9]. While mitigation efforts implemented to curb the spread of the pandemic were well intended, these measures may have restricted access to activities offering benefits for physical and mental health and well-being, highlighting the need to evaluate efforts to facilitate access to health-promoting activities in the context of the pandemic.

Hiking can be defined as “walking for a substantive distance in the outdoors, often over natural terrain with obstacles such as rocks and tree roots to navigate around” [10]. Prior to the COVID-19 pandemic, hiking had been highlighted as a promising way to address sedentary lifestyles, given its low cost, accessibility to many, and variable levels of difficulty [10]. In addition, hiking fits with pandemic-era recommendations to reduce COVID-19 transmission by choosing activities that can take place outdoors, away from crowds [11]. Facilitating access to hiking is also expected to benefit various aspects of health and well-being, given links between outdoor physical activity and various physical and mental health outcomes [1–4, 10, 12]. Previously, physical activity [13] and exposure to green space [14] have been linked with improved sleep quality and quantity, and outdoor physical activities have been linked with decreased stress [4]. Taken together, it follows that increasing access to outdoor physical activity has the potential to attenuate the decreased physical activity, increased sedentary activity [15], poorer sleep, and increased stress [5–7] observed among adults during the COVID-19 pandemic. Impacts on these variables would be meaningful, as each of them has been shown to impact physical and mental health over time (e.g., [16]).

Compared to sedentary exposure to nature or indoor physical activity, there is some evidence of greater positive effects of physical activity in nature, including positive impacts on emotions and decreased fatigue and stress [4, 17]. Given the recency of the COVID-19 pandemic, there is limited intervention research examining the potential of outdoor physical activity to have health benefits in the context of the pandemic specifically. However, the small body of research that exists is consistent with the general idea that promoting physical activity in nature could have multiple physical and mental health benefits. For example, in a recent longitudinal study of the “Moving Parks” project, which provided free physical activities in public parks in Italy, there were significant improvements in anxiety, depressed mood, self-control, well-being, and vitality in a sample of adults from before to after 3 months of exercise in nature during Spring/Summer 2021 [18]. These findings are promising, although causality cannot be determined in the absence of a randomized design.

The Get Outside Study was created to examine whether providing free access to a regional winter hiking challenge would impact adults’ activity choices, including how often they went hiking, and also promote aspects of their well-being, including increased sleep duration and decreased stress, during the COVID-19 pandemic. The initial, randomized Get Outside Study was conducted from January to March 2021, about one year into the COVID-19 pandemic. We conducted a second randomized study with a separate sample of participants one year later in Winter 2022. In addition to testing effects of this hiking intervention on key outcomes of hiking frequency, sleep duration, and stress, we also: (1) monitored aspects of intervention implementation, including engagement in the winter hiking challenge; (2) assessed whether the intervention affected the frequency of other types of activities, including physical and social activities and activities expected to confer lower or higher risk for COVID-19 transmission (in Study 1); and (3) explored potential intervention effects on an additional aspect of sleep, sleep quality (in Study 2).

## Study 1 methods

### Participants

A convenience sample of adults (n = 53) was recruited in January 2021 using online methods, including posted advertisements on social media and emails sent to a database of individuals who had previously indicated interest in participating in research. Recruitment advertisements specified that participants would be asked to complete 3 online surveys during the 3-month study and would receive activity ideas and resources, including hike/nature walk locations and maps, access to social media groups, and the chance to earn prizes. Eligibility criteria included adults 18 years or older who were English speaking; had online access; lived in the Western New York region of the United States; were interested in receiving suggestions for ways to get outside, stay active, and stay busy during COVID-19; had no health problems precluding participation; and were not currently involved in regular (i.e. at least weekly) hikes or nature walks. Participants were ineligible if they did not meet these criteria, including having any health problems that would make it difficult for them to hike (e.g., recent surgery) or living outside of the counties of Western New York. 346 individuals completed an eligibility survey, 185 of whom were eligible. Fifty-three participants completed baseline procedures described herein and then were randomized to the intervention or control group, at which point the target sample size was reached, and enrollment ceased. Allocation to study groups was nearly equal (49% intervention). Mid-point surveys were sent to participants about 6 weeks after enrollment, and post-intervention surveys were sent about 5 weeks after that, at the end of the winter hiking challenge. The 41 participants who engaged in study procedures after baseline (i.e. by completing at least one additional study survey after baseline) were included in primary data analyses; we repeated analyses to examine whether results were consistent using all available data from all 53 randomized participants. Demographic characteristics of these samples are in Table 1, and the CONSORT participant flow diagram is in Electronic Supplementary Material 1.

**Table 1:** Study 1: Demographic characteristics of participants at baseline

	Mean ± SD or Frequency (%)	
	Randomized sample (n = 53)	Analytic sample (n = 41)
Sex	92.5% female, 7.6% male	92.7% female, 7.3% male
Age	47.8 ± 10.6 years	48.3 ± 11.1 years
Race/ethnicity	94.3% white, 3.8% Black, 1.9% Asian and white	95.1% white, 4.9% Black
Education	5.6% Associate’s degree, 37.7% BA/BS, 56.6% graduate degree	7.3% Associate’s degree, 39.0% BA/BS, 53.7% graduate degree
Marital status	77.4% married, 9.4% single, 7.6% living with partner, 5.7% divorced	73.2% married, 12.2% single, 9.8% living with partner, 4.9% divorced
Annual household income	3.7% <$10,000, 18.9% $50,000-$74,999, 22.6% $75,000-$99,999, 35.9% $100,000-$149,999, 9.4% >$150,000, 9.4% prefer not to answer	4.9% <$10,000, 19.5% $50,000-$74,999, 19.5% $75,000-$99,999, 36.6% $100,000-$149,999, 7.3% >$150,000, 12.2% prefer not to answer
Total individuals in household	3.4 ± 1.4	3.1 ± 1.3
Children ≤ 18 years in household	45.2% none, 18.9% one, 22.6% two, 13.2% three	51.2% none, 19.5% one, 22.0% two, 7.3% three

Note: BA/BS = Bachelor’s degree. Only categories that were endorsed are presented (e.g., no participant reported an income between $10,000-$49,999). In cases where percentages do not total 100, this is due to rounding. The analytic sample for primary analyses was all participants who engaged with study procedures at least once after baseline

### Procedures

Participants completed online surveys following recruitment (baseline) and ~ 6 (midpoint) and ~ 11 weeks after baseline (post-intervention). Surveys assessed demographics, usual activities, stress, sleep, and experiences with the COVID-19 pandemic, as detailed below. Within days of baseline survey completion, participants were randomized to a study group using a parallel design and random number sequence generated in Microsoft Excel by the study team member without direct participant contact (SAF). Upon randomization, the intervention group received immediate, complimentary access to the Western New York Winter Hiking Challenge, a regional hiking challenge that was organized by Outside Chronicles (https://outsidechronicles.com/) and ran from late December 2020 through late March 2021. Participants received a code allowing them to register for this hiking challenge for free ($20 value), and upon registering, received access to the hiking challenge materials, which included a list of local hikes, corresponding digital maps, membership in social media groups, and the opportunity to earn a sticker and patch for completing 8 or more indicated hikes. In an effort to match study timelines and incentives across study groups, the control group was also provided with materials. First, upon randomization, the control group received an activity sheet with ideas to stay busy during COVID-19 (e.g., virtual paint night, stargazing), with links to corresponding instructions and resources. The provision of the activity sheet matched the timing of the intervention-group’s receipt of hiking challenge materials. The control group also received a delayed intervention after the completion of all study surveys, in order to provide equally valued materials to the two groups during the study. Specifically, the control group received access to the Western New York Summer Hiking Challenge after completion of the post-intervention survey, as this was the hiking challenge that was beginning at the time. After post-intervention survey completion, the intervention group also received the study activity sheet, so that over the course of the study, each group received access to both an activity sheet and a hiking challenge, with the intervention of interest (access to a hiking challenge) provided to the control group after all study measures were completed, in order to avoid contamination. Study procedures for this study as well as Study 2 were reviewed and approved as exempt by the University at Buffalo Institutional Review Board and registered at clinicaltrials.gov prior to participant enrollment.

### Measures

Online surveys were completed at baseline, midpoint, and post-intervention, with specific measures detailed below. Each measure was collected at all time points unless specified. As indicators of intervention implementation, participants indicated whether they completed intervention activities and how much they liked them at midpoint and post-intervention. The intervention group reported whether they signed up for the hiking challenge, completed challenge hikes, and if applicable: who they hiked with, how difficult hikes were, how enjoyable hikes were, whether they completed the challenge, and whether they planned to continue hiking. There was also an open-ended question inviting participants to share any other details that they would like to share about their experiences with the winter hiking challenge. The control group completed similar questions about the provided activity sheets and also reported on whether they had heard of and engaged in the Western New York Winter Hiking Challenge at post-intervention, as an indicator of potential contamination between study groups.

Primary outcome: Frequencies of activities of interest during the past month. An adapted version of the Pleasant Activities List [19] was used to assess the frequency that participants engaged in a list of 91 different activities from never in the past 30 days (0) to more than once per week (4), as well as whether the activities were done: (1) indoors or outdoors and (2) alone, with others from the household, or with others not from the household. Participants were asked, “please rate how often, where, and with whom you did each activity in the past 30 days”, with each activity (e.g., reading, going for a hike or nature walk) listed and opportunities to indicate frequency, location, and companions for each. We started with the existing list of 139 activities, trimming it for parsimony (e.g., merging a few activities that were similar to one another and not of primary interest, such as keeping an aquarium or a terrarium with exotic animals, also deleting a few items that we expected to be completed infrequently and that were not of primary interest, such as going bungee jumping). We then added or rephrased a few additional items in order to ensure fit with our primary study aims and the current context (e.g., modifying an item that included hiking, camping, and other outdoor activities to focus on hiking and nature walks specifically). The 5-point frequency scale used was from the original measure; we added the questions about activity location and companions. The adapted version of this measure is available from the authors upon request.

Outcome variables from this measure included the primary outcome of how often participants hiked/went on nature walks, as well as how often participants engaged in activities expected to be lower vs. higher risk for COVID-19 and how often participants engaged in social and physical activities. Following guidance from the Centers for Disease Control and Prevention, we operationalized lower-risk activities as activities completed outdoors as well as those completed indoors with members of one’s household or alone, summing across the frequencies of each applicable activity to arrive at a total low-risk activity frequency score. Higher-risk activities were those completed indoors with individuals who do not live in one’s household, with sum scores calculated for these. Frequencies of physical and social activities were also created by summing the frequencies of all items in each of these categories, with categorization adapted from prior research [20].

Secondary outcomes: Stress and sleep duration during the past month. The 10-item, validated Perceived Stress Scale [21] was used to assess participants’ stress the prior month. Participants rated how often they felt a certain way (e.g., upset because of something that happened unexpectedly) on a scale of 0 (never) to 4 (very often). A total score was calculated from the items, with higher scores indicating greater stress. An item from the Pittsburgh Sleep Quality Index [22] was used to assess nightly sleep duration (“During the past month, how many hours of actual sleep did you get at night?”).

Demographics. Participants completed demographic survey questions about themselves (e.g., age, race/ethnicity, sex, education level) and their household (e.g., number of adults and children in the household, income) at baseline.

COVID-19 experiences. Participants completed questions about whether anyone close to them tested positive for COVID-19 in the past month and ways the COVID-19 pandemic impacted them over the past month (e.g., changes to employment, financial challenges) [23]. These questions were included to contextualize participants’ experiences at the time of the study, similar to other descriptive information collected, such as demographics.

### Data analysis

Distributions for all variables of interest were examined. Descriptive statistics were calculated to summarize participant demographics, COVID-19 experiences, stress, and sleep at baseline, as well as intervention implementation. Frequencies were conducted on categorical variables and means on continuous variables. We conducted bivariate analyses to test for study group differences in demographics and behaviors of interest at baseline. Following these analyses, we considered household size as a covariate. We also examined inter-correlations between behavioral variables of interest.

Repeated measures ANOVA models were conducted to test intervention effects on outcome variables of interest. The primary models incorporated all available data from the aforementioned sample of 41 participants, examining the group effect, time effect, and group-by-time interaction in predicting key outcomes (frequency of hiking, nightly sleep duration, and stress scores) and other, ancillary outcomes of interest assessed in this study (high-risk activities, low-risk activities, physical activities, and social activities). Here participants were grouped based on assigned study groups, regardless of intervention engagement. We examined the aforementioned main effects and interactions, adjusting for total household size, and conducted a priori planned contrasts, examining study group differences in least squares means for each outcome at midpoint and at post-intervention.

After primary analyses, we repeated these models twice: first in the full sample of all 53 randomized participants (12 of whom did not engage with study activities after baseline) and again with engagement in the intervention, rather than assigned study group, as the predictor of interest. The latter was a post-hoc analysis, which was added after noting that only 10 intervention-group participants engaged in the hiking challenge by completing challenge hikes, and hypothesizing that impacts of this intervention might be greater with increased engagement. We confirmed completion of challenge hikes for one control group participant who found out about the challenge on their own. We considered this individual, plus the 10 intervention group participants who completed challenge hikes, as the group actively engaged in the winter hiking challenge. We examined engagement as a predictor of outcomes of interest to inform the potential of conducting a replication study designed to increase uptake of the intervention and reassess its impacts on outcomes.

## Study 1 results

### Descriptive statistics

COVID-19 experiences, stress, and sleep. Participants’ reported experiences with COVID-19 at baseline are shown in Table 2. The most commonly reported COVID-19 pandemic impacts were working from home more, working while a child or children were home with them, and a friend or acquaintance testing positive for COVID-19. Participants had an average score of 16.3 ± 6.4 on the Perceived Stress Scale (possible range = 0–40) at baseline and reported sleeping an average of 6.7 ± 1.1 h per night.

**Table 2:** COVID-19 related experiences of participants in Study 1

	Mean ± SD or Frequency (%)	
	Randomized sample (n = 53)	Analytic sample (n = 41)
*Has anybody close to you tested positive for COVID-19 in the past month?*		
Yes, I have	3.8%	2.4%
Yes, at least 1 family member	28.3%	24.4%
Yes, at least 1 friend/acquaintance	43.4%	43.9%
*Experienced any of the following as a result of COVID-19 in the past month?*		
Worked from home more than usual	54.7%	53.7%
Worked more hours than usual	17.0%	12.2%
Worked reduced hours	11.3%	14.6%
Was not able to work due to COVID-19 illness	5.7%	4.9%
Became unemployed	3.8%	4.9%
Difficulty arranging childcare	5.7% (10.3% of those with children)	4.9% (10.0%)
Worked with children at home with me	28.3% (51.7% of those with children)	23.4% (45.0%)
Income or pay reduced	13.2%	12.2%
Not paid at all	0.0%	0.0%
Not enough money for gas	1.9%	0.0%
Not enough money for food	3.8%	2.4%
Serious financial problems	0.0%	0.0%

Impacts no participants endorsed across Studies 1 and 2 were: increased childcare costs, not enough money for rent or medications, no regular place to sleep.

When examining inter-correlations between key behaviors of interest in the analytic sample, hiking frequency, stress, and sleep were generally linked as expected, although these links tended to be stronger at later study time points. Greater hiking frequency was linked with more hours of sleep per night at midpoint at a trend level (r = 0.31, p = 0.06), and this relationship was statistically significant post-intervention (r = 0.43, p < 0.01). There was no association at baseline. Greater hiking frequency was linked with less stress post-intervention (r=-0.36, p < 0.05). The magnitude of this correlation was in the same direction at the other time points but not statistically significant. These relationships were generally consistent with and without an adjustment for study group and when including all enrolled participants.

Intervention implementation. Overall, 22 of 26 (84.6%) participants randomized to the intervention group signed up for the Western New York Winter Hiking Challenge. Of those who signed up, 45.5% reported completing at least 1 challenge hike, and 18.2% completed the challenge by completing 8 hikes. Those completing challenge hikes at midpoint reported hiking with spouses/partners, children, other family members, and friends, and generally found hikes to be enjoyable and of an appropriate difficulty. At post-intervention, results were similar, with full intervention implementation data at post-intervention shown in Table 3. Open-ended comments about the winter hiking challenge included positive comments that the challenge encouraged participants to get outside in the winter and helped improve mood, as well as enjoyment of maps and information provided and plans to try more of the hikes in the future. The most commonly reported barrier to implementation was a lack of supplies or equipment for winter hiking, with other barriers relating to a lack of comfort level or ability to hike in the winter.

**Table 3:** Intervention implementation and acceptability in Study 1

	Mean ± SD or Frequency (%)
	Among those randomized to intervention (n = 26)
Signed up for Winter Hiking Challenge	22 (84.6% of those randomized)
*Of those who signed up for the Winter Challenge after being provided with access as part of the study*:	
Reported completed any challenge hikes	10 (45.5% of those who signed up)
Completed the Winter challenge (8 hikes)	4 (18.2% of those who signed up)
Signed up for the next (Summer) challenge	5 (50.0% of those engaged in the Winter challenge)
*Perspectives at post-intervention among intervention group participants completing any challenge hikes*:	
Completed challenge hikes with (select all that apply):	10.0% self, 80.0% spouse/partner, 40.0% children, 10.0% other family, 10.0% friend, 20.0% dog
How enjoyable were challenge hikes?	0.0% not enjoyable, 20.0% moderately enjoyable, 80.0% very enjoyable
How difficult were challenge hikes?	10.0% a little bit easy, 80.0% just right, 10.0% a little bit hard
Do you plan to continue hiking regularly?	50.0% yes via another challenge, 40.0% yes not in a challenge, 10.0% no

We were able to confirm that one control-group participant completed winter challenge hikes via records from the hiking challenge (i.e. this participant was randomized to the control group but found out about and engaged in the hiking challenge on their own). After post-intervention, when free registration for the Western New York Summer Challenge was provided to the control group, 12 control-group participants signed up for this hiking challenge. In addition, 5 intervention-group participants signed up for the Summer Hiking Challenge on their own, indicating maintenance among half of the intervention-group participants who were active in the winter challenge.

### Intervention effects on activities

There were no significant intervention effects on activity variables of interest in the analytic sample (n = 41), including the key outcome of hiking frequency (p = 0.66), as well as frequencies of high-risk, low-risk, physical, or social activities (p ≥ 0.14). There were also no statistically significant time effects or group-by-time interactions, nor were there significant differences between the two study groups at individual time points. Total household size was a statistically significant predictor in some models, such that larger households engaged in high-risk activities less frequently, low-risk activities more frequently, and social activities more frequently (p < 0.05 for each). Results were generally consistent when repeating these analyses using all available data from all randomized participants (n = 53), with the one difference being that there was a trend-level group difference in the frequency of physical activities in this model, such that the intervention group tended to do physical activities more often than the control group overall (p = 0.07).

### Intervention effects on sleep duration and stress

**Fig. 1A-1F:**
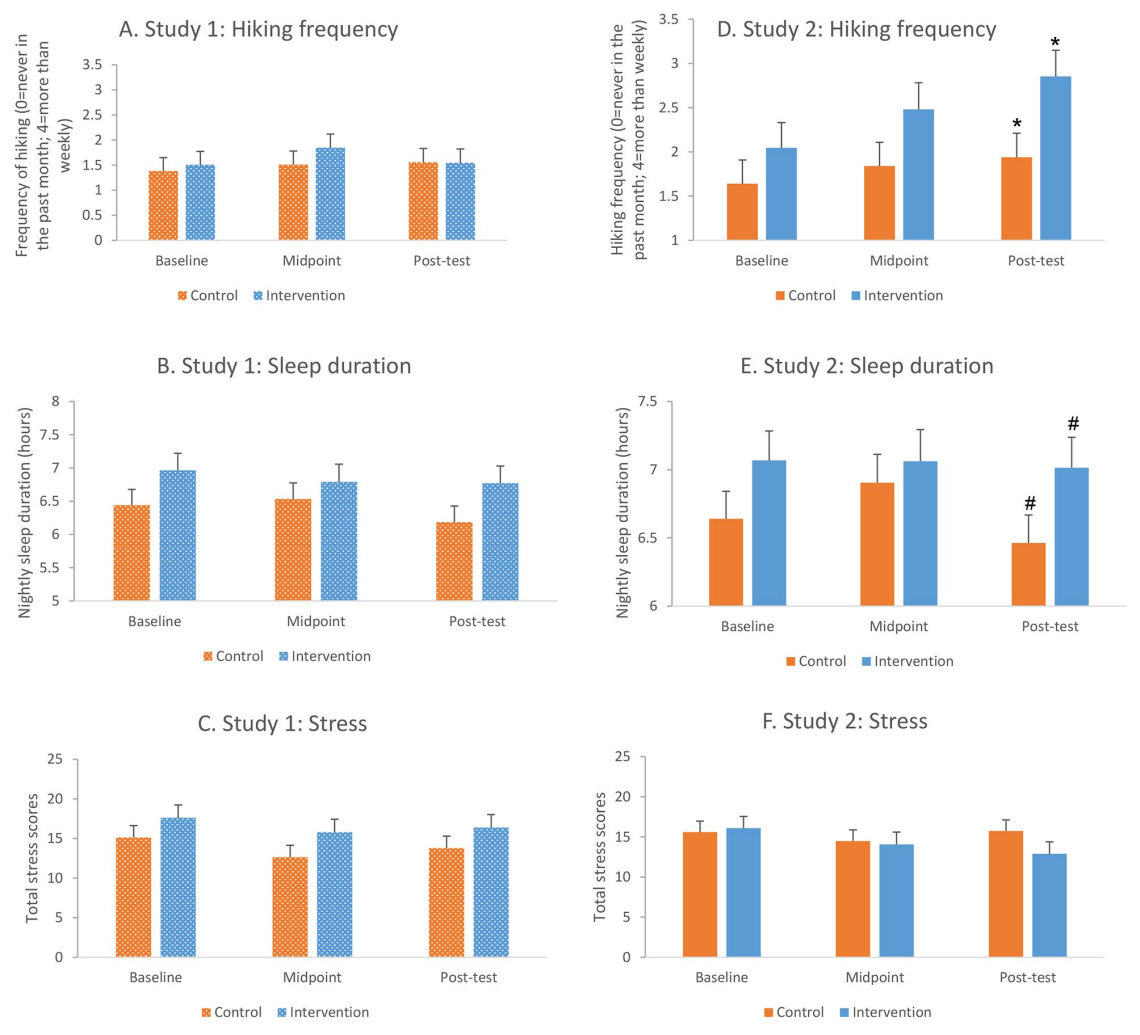
**Effects of study group on main outcomes of interest in Study 1 (n = 41) and Study 2 (n = 47).** While some group differences were in the expected direction, there were no overall significant intervention effects on key outcomes of interest in Study 1, nor were any of the comparisons of least squares means at individual time points significant. In Study 2, in which winter traction cleats were provided to address barriers, and engagement in the intervention was higher, results were in the expected direction for all outcomes. The intervention group hiked more frequently than the control group, with statistically significant overall group effects and between-group differences at post-intervention (p < 0.05), and there was a trend toward increased nightly sleep duration by post-intervention (p = 0.07). Cohen’s d values for the group differences at post-intervention in Study 2 are: hiking frequency d = 0.67, sleep duration d = 0.55, stress d = 0.42. Values shown are least squares (LS) means and standard errors from repeated measures models. Results were similar when repeating analyses in all randomized participants (n = 53; n = 51). Symbols indicate that LS means at the indicated time point differed between groups at *p < 0.05; #p < 0.10

There were no significant intervention effects on sleep duration, nor were there significant changes over time or group-by-time interactions in predicting sleep (p ≥ 0.17). There were also no intervention effects on stress, but there was a significant change in stress over time (p < 0.01), such that the magnitude of stress scores decreased from baseline to midpoint and then increased again. This pattern did not differ by group. There were no significant differences in either of these outcomes when comparing the two study groups at individual time points, and household size was not a significant predictor of outcomes in either of these models. These results were generally consistent when repeating the analyses with all randomized participants, although in the model predicting stress, the relationship between total household size and stress became significant (p < 0.05), such that larger household size was linked with greater stress. Figure 1 A-1 C depict least squares means for key variables of interest over time by study group.

### Post-hoc analyses examining engagement in the intervention as the predictor

After noting that only 38.5% of participants who were randomized to the intervention group engaged in the hiking challenge by completing any challenge hikes, and hypothesizing that impacts of this intervention might be greater with increased engagement, we conducted an exploratory, post-hoc analysis comparing those who engaged in the challenge (10 in the intervention group plus the 1 control group participant who engaged in the challenge on their own) to those who did not. Overall, those engaged in the challenge had hiked significantly more often than those not engaged (F = 7.82, p < 0.01). Results were similar when examining frequency of physical activities generally: the link between engagement and physical activity frequency did not reach statistical significance overall (p = 0.06), but examination of least squares means at each time point showed a statistically significant difference by post-intervention, with more physical activity in the engaged group (p < 0.05). Engagement in the hiking challenge was not a significant predictor of the other outcomes of interest, although group differences between those engaged in the winter hiking challenge vs. the rest of the sample were in the expected direction for high-risk and low-risk activities and sleep. Most baseline variables were not significantly linked with engagement, although those who engaged in the challenge did already hike more often at baseline, highlighting that additional research was needed to understand potential impacts of engaging in this intervention.

## Study 2 methods

### Participants

To continue to study the potential impact of this intervention, we conducted another randomized study with a similar timeline and methods, recruiting a new convenience sample of adults in January 2022 with recruitment methods and eligibility criteria in parallel to Study 1. 207 individuals completed an eligibility survey, 162 of whom were eligible. Fifty-one participants completed baseline procedures and were randomized to a study group, at which point the target sample size was reached, and enrollment ceased. Forty-seven participants were included in the analytic sample of those who engaged with at least one study activity beyond baseline (i.e. the midpoint survey sent ~ 6 weeks after enrollment and/or the post-intervention survey sent about 6 weeks later). Demographic characteristics of participants are in Table 4, and the CONSORT participant flow diagram is shown in Electronic Supplementary Material 2.

**Table 4:** Study 2: Demographic characteristics of participants at baseline

	Mean ± SD or Frequency (%)	
	Randomized sample (n = 51)	Analytic sample (n = 47)
Sex	90.2% female, 9.8% male	89.4% female, 10.6% male
Age	47.2 ± 12.2 years	46.9 ± 12.4 years
Race/ethnicity	96.1% white, 3.9% Hispanic/ Latino, 2.0% Middle Eastern	95.7% white, 4.3% Hispanic/Latino, 2.1% Middle Eastern
Education	17.7% Associate’s degree or some college, 27.5% BA/BS, 54.9% graduate degree	19.2% Associate’s degree or some college, 25.5% BA/BS, 55.3% graduate degree
Marital status	68.6% married, 15.7% single, 2.0% living with partner, 7.8% divorced, 3.9% separated, 2.0% other	68.1% married, 17.0% single, 2.1% living with partner, 6.4% divorced, 4.3% separated, 2.1% other
Annual household income	2.0% $10,000-$14,999, 3.9% $25,000-$34,999, 7.8% $25,000-$49,999, 21.6% $50,000-$74,999, 5.9% $75,000-$99,999, 37.3% $100,000-$149,000, 15.7% $150,000 or more, 5.9% prefer not to answer	2.1% $10,000-$14,999, 4.3% $25,000-$34,999, 8.5% $25,000-$49,999, 23.4% $50,000-$74,999, 6.4% $75,000-$99,999, 36.2% $100,000-$149,000, 12.8% $150,000 or more, 6.4% prefer not to answer
Total individuals in household	3.0 ± 1.4	3.0 ± 1.5
Children ≤ 18 years in household	52.9% none, 15.7% one, 15.7% two, 15.7% three	53.2% none, 17.0% one, 12.8% two, 17.0% three

Note: BA/BS = Bachelor’s degree. Only categories that were endorsed are presented. In cases where percentages do not total 100, this is due to rounding, or being able to select multiple categories, in the case of race/ethnicity. The analytic sample for primary analyses was all participants who engaged with the study at least once after baseline

### Procedures

The design of this replication study was nearly identical to Study 1 with two changes: (1) we provided seasonally-appropriate hiking equipment based on barriers reported in the first study; (2) we made minor modifications to the online surveys as described below. Collection of priority outcomes (hiking frequency, sleep duration, stress) remained the same as Study 1. We set out to examine whether we could increase uptake of this intervention by addressing reported barriers, and whether this would impact hiking frequency and aspects of well-being as well. Because a substantial number of participants in Study 1 reported barriers related to equipment for and/or comfort with winter hikes, in Study 2 we mailed winter traction cleats to the intervention group after randomization in an effort to increase the feasibility of the challenge hikes.

Participants completed online surveys following recruitment (baseline) and ~ 6 (midpoint) and ~ 12 weeks after baseline (post-intervention). Participants were randomized to a study group after baseline as in Study 1. Following randomization, the intervention group received access to that year’s Western New York Winter Hiking Challenge, which ran from late December 2021 through late March 2022. Participants received a code that allowed them to register for this hiking challenge for free ($20 value), and upon registering, received access to hiking challenge materials as in Study 1. In this study, they also received winter traction cleats shortly after randomization via postal mail. The timing and content of the activity sheet and delayed intervention provided to the control group were the same as in Study 1, plus the control group also received hiking socks at the end of this study that were of a similar monetary value as the winter traction cleats, in an effort to balance the materials provided across groups over the course of the study.

### Measures

Key primary (hiking frequency) and secondary outcome (stress, sleep duration) measures were consistent with Study 1, as were measurement of demographics and intervention implementation. Each Study 2 measure described below was collected at baseline, midpoint, and post-intervention unless specified. Any small changes to online surveys between Study 1 and 2 are described.

Primary outcome: Frequency of hiking/nature walks. An adapted version of the Pleasant Activities List [11] was used to assess the frequency that participants engaged in 10 different activities from never in the past 30 days (0) to more than once per week (4) and whether the activities were done: (1) indoors or outdoors and (2) alone, with others from the household, or with others not from the household. The Pleasant Activities List was shortened in this study compared to the version administered in Study 1 in order to address participant comments about the length of the survey while still allowing consistent assessment of the primary outcome (how often participants hiked/went on nature walks).

Secondary outcomes: Stress and sleep. The same items from Study 1 were used to assess stress and sleep duration. In addition, another question from the Pittsburgh Sleep Quality Index [22] was added to midpoint and post-intervention surveys in Study 2 to assess self-reported sleep quality over the past month, with possible responses of very good, fairly good, fairly bad, and very bad.

Other variables. Participants completed questions about whether anyone close to them tested positive for COVID-19 and ways the pandemic impacted them over the past month [23]. In Study 2, COVID-19 questions were part of the midpoint survey rather than the baseline survey. We had originally planned to reduce the COVID-19 questions, but with the emergence of the Omicron variant, we decided to reintroduce our complete set of COVID-19 questions at midpoint to provide context given anticipated continued impacts of the COVID-19 pandemic on participants’ lifestyles.

### Data analysis

Data analyses were generally the same as in Study 1, including descriptive statistics, inter-correlations, examination of intervention implementation, and the use of repeated measures ANOVAs to examine intervention effects on hiking frequency, stress, and sleep duration, with primary models including all available data from all participants who engaged with any study activities after baseline (n = 47). We repeated analyses to see whether primary results were consistent when: adjusting for household size, repeating analyses for all randomized participants (n = 51), and operationalizing the predictor as those who engaged in the intervention versus those who did not, rather than assigned study group. Finally, in this study, we also explored study group differences in sleep quality at midpoint and post-intervention.

## Study 2: results

### Descriptive statistics

Sociodemographics, COVID-19, stress, and sleep. There were no differences between groups in baseline sociodemographics. Reported experiences with COVID-19 are in Table 5. Participants had an average score of 15.8 ± 7.1 on the Perceived Stress Scale at baseline and reported sleeping an average of 6.8 ± 1.2 h per night. There were no significant cross-sectional links between hiking frequency, stress, and sleep duration in this sample.

**Table 5:** COVID-19 related experiences of participants in Study 2

		Mean ± SD or Frequency (%) Experiences at midpoint* (n = 41)	
*Has anybody close to you tested positive for COVID-19 in the past month?*			
Yes, I have		12.2%	
Yes, at least 1 family member		14.6%	
Yes, at least 1 friend/acquaintance		31.7%	
*Experienced any of the following as a result of COVID-19 in the past month?*			
Worked from home more than usual		4.9%	
Worked more hours than usual		4.9%	
Worked reduced hours		17.1%	
Was not able to work due to COVID-19 illness		12.2%	
Became unemployed		0.0%	
Difficulty arranging childcare		0.0%	
Worked with children at home with me		2.4% (5.0% of those with children)	
Income or pay reduced		2.4%	
Not paid at all		2.4%	
Not enough money for gas		0.0%	
Not enough money for food		0.0%	
Serious financial problems		2.4%	

Impacts no participants endorsed across Studies 1 and 2 were: increased childcare costs, not enough money for rent or medications, no regular place to sleep. *In Study 2, COVID-19 related experience questions were asked at midpoint rather than baseline, as described in the text

Intervention implementation. Twenty-two of 25 participants randomized to the intervention group signed up for the Western New York Winter Hiking Challenge. Of those who signed up, 54.5% completed at least 1 challenge hike, and 31.8% completed the challenge. Implementation data are in Table 6. Open-ended comments about the winter hiking challenge included positive comments (the challenge encouraged participants to get outside/get out, looked forward to planning hikes, enjoyed/loved the challenge, appreciated the winter traction cleats, lost weight), as well as barriers to implementation related to travel logistics, health/COVID-19, time, and to a lesser extent, a lack of knowledge and proper gear. Again, 1 control-group participant found out about and engaged in the winter hiking challenge on their own. Thirteen control-group participants signed up for the Western New York Summer Hiking Challenge provided to them as a delayed intervention, and 7 intervention-group participants signed up for the summer challenge, indicating maintenance among more than half of the intervention-group participants who had been engaged in the winter challenge.

**Table 6:** Intervention implementation and acceptability in Study 2

	Mean ± SD or Frequency (%)
	Among those randomized to intervention (n = 25)
Signed up for Winter Hiking Challenge	22 (88.0% of those randomized)
Used winter traction cleats provided	14 (70.0% of intervention participants completing post surveys reported use)
*Of those who signed up for the Winter Challenge after being provided with access as part of the study*:	
Reported completed any challenge hikes	12 (54.5% of those signed up)
Completed the Winter challenge (8 hikes)	7 (31.8% of those signed up)
Signed up for the next (Summer) challenge	7* (58.3% of those engaged in the Winter challenge)
*Perspectives at post-intervention among intervention group participants completing any challenge hikes*:	
Completed challenge hikes with (select all that apply):	16.7% self, 50.0% spouse/partner, 25.0% children, 8.3% other family, 50.0% friend, 8.3% dog, 8.3% other (women’s group)
How enjoyable were challenge hikes?	0.0% not enjoyable, 16.7% moderately enjoyable, 83.3% very enjoyable
How difficult were challenge hikes?	75.0% just right, 25.0% a little bit hard
Do you plan to continue hiking regularly?	58.3% yes via another challenge, 41.7% yes not in a challenge

*To date (the 2022 Western New York Summer Challenge was ongoing at the time of this writing)

Intervention Effects on Hiking Frequency. There was a significant overall intervention effect on hiking frequency in the analytic sample (n = 47), such that the intervention group hiked more often than the control group overall (F = 4.23, p < 0.05; Cohen’s d = 0.60). There was also a significant change in hiking frequency over time (F = 3.63, p < 0.05), but no significant group-by-time interaction (p = 0.47). Group differences in least squares means at individual time points were not statistically significant at baseline or midpoint, with Cohen’s d values of 0.29 and 0.48 respectively, but were statistically significant by post-intervention (Fig. 1D; d = 0.67). Results were generally the same when repeating these analyses adjusting for total household size and in the 51 randomized participants, although the overall group effect on hiking frequency no longer reached statistical significance in the latter model.

Intervention Effects on Sleep Duration, Sleep Quality, and Stress.

There were no overall intervention effects on sleep duration (p = 0.12). However, overall mean differences were in the expected direction, corresponded to a Cohen’s of 0.45 overall, and reached trend level by post-intervention, such that the intervention group tended to have longer nightly sleep durations than the control group by the end of the study (p = 0.07; d = 0.55; Fig. 1E). There were no main effects of time or group-by-time interactions in the model predicting sleep duration. Results were similar when adjusting for total household size and when repeating the model among all 51 randomized participants, with the overall group difference in nightly sleep duration reaching a trend level (p < 0.10) in the former analysis. The ancillary analysis examining sleep quality was consistent with these findings, such that there were not significant differences in self-reported sleep quality at midpoint (p = 0.10), but by post-intervention, the intervention group reported significantly better sleep quality than controls (t(42) = 2.3, p < 0.05).

With regards to stress, there were no statistically significant group, time, or group-by-time interactions, nor were the between-group comparisons of least squares means at each time point statistically significant, but magnitudes of the mean differences were in the expected direction, with increases over time (d = 0.07, d = 0.17, and d = 0.42 for between-group comparisons at baseline, midpoint, and post-intervention, respectively; Fig. 1F). Results were consistent when repeating this model adjusting for household size and with all randomized participants. Generally, group differences in key outcomes became stronger when operationalizing the predictor as engagement in the intervention as opposed to study group assignment, although sleep duration was an exception to this finding.

## Discussion

Overall, results from this pair of studies are consistent with hypotheses that this intervention has the potential to promote increased hiking frequency, as well as other aspects of health and well-being that have been linked with outdoor physical activity. In Study 2, winter traction cleats were provided to the intervention group to address barriers reported in the first study, in which engagement in the intervention was relatively low, but links between hiking frequency and well-being outcomes suggested the potential of the intervention with greater uptake. Engagement was higher in the second study, and there were significant intervention effects on hiking frequency and evidence of improvements in sleep in the intervention group by post-intervention. While effects on stress did not reach statistical significance, results were in the expected direction.

Addressing the main barrier to intervention engagement reported in Study 1 seemed to improve intervention uptake in Study 2, yet just under half of the participants assigned to the intervention group still did not engage in any challenge hikes. In Study 2, a lack of winter hiking equipment or comfort hiking outdoors in the winter were no longer mentioned as common barriers, but some new barriers emerged as themes, including challenges with traveling to the hiking locations and also health-related barriers such as testing positive for COVID-19. The former is an important barrier to note, given the goal to make interventions like this accessible to all individuals. The majority of participants in both of these studies were white and middle-to-upper-income. Addressing barriers such as the financial and time costs of traveling to hiking locations across the region may broaden accessibility of this intervention, including to those from lower-income households. The organizer of this regional challenge has since debuted an Urban Hiking Treks series, which features a set of walks that can be completed outdoors within a shorter radius around Buffalo, New York, perhaps offering one way to address this barrier among those who live in and around this city.

Health-related barriers are also notable and may have been especially problematic during Winter 2022 with the emergence and rapid escalation of the Omicron variant of COVID-19. In examining public records, we noted a greater prevalence of COVID-19 cases in Winter 2022 versus 2021 in the region under study, as well as a colder, snowier winter in 2022. For example, in aggregating daily weather data from the National Oceanic and Atmospheric Administration from January-March 2021 and 2022 across the 5 counties of the Western New York region, we found that the average low temperature was 5 degrees colder in 2022 compared to 2021, and that the total inches of snowfall was 69.0 inches in 2022 versus 33.6 in 2021. In addition, within the study data, the percent of individuals reporting a recent positive COVID-19 test was higher in Study 2 versus Study 1. It is notable that engagement in the intervention increased from Winter 2021 to 2022, even with these potential health and weather-related barriers. Participants randomized to the intervention group in both studies had control over whether and when they hiked, offering more flexibility than many existing studies testing effects of outdoor physical activity, in which study participants visit the researchers to participate in assigned physical activity. While this flexibility may have affected implementation, it is also a strength in terms of the ecological validity of this intervention and its potential for sustainability in the context of participants’ everyday lives.

The present studies have implications beyond the COVID-19 context. The hiking interventions were implemented in the winter in the Western New York region of the United States, which is known for cold and snowy weather. While pandemics may represent one context in which physical activity decreases, sedentary activity increases, and associated health outcomes worsen, winter is another time in which individuals may be less likely to engage in physical activity outdoors. Continuing to build upon the present study findings offers potential to promote outdoor physical activity and associated health benefits during times in which these activities tend to decline. Given the demonstrated health benefits of outdoor physical activity (e.g., [4, 17]), positive outcomes linked with this intervention may extend beyond those outcomes measured here. Exploratory analyses that were conducted during Study 2 to inform future research support this point, with evidence of medium-sized effects of this intervention on participants’ self-reported interconnectedness with nature and subjective vitality at post-intervention (data not shown).

Limitations of the present studies included the small samples, which as mentioned above, were fairly homogeneous demographically. These limitations impact the generalizability of the present findings, and it is not yet known whether results would be consistent (or weaker or stronger) in larger, more diverse samples. Initial findings across these two studies suggest that increasing uptake of this intervention in the present samples is linked with increased hiking, as well as other well-being benefits. While effects were generally in the expected direction across outcomes measured in Study 2, some did not reach statistical significance, with the small samples potentially constraining the ability to detect effects. Future research can examine whether effects are augmented when recruiting larger, more diverse samples and continuing to address reported barriers to improve uptake. Previous reviews have noted the potential for links between outdoor recreation and health benefits to be even greater among low-income populations, further highlighting the potential of bringing a feasible, acceptable version of this intervention to populations at disproportionate risk for negative health outcomes [12]. Other ways to diversify the sample would be to aim for more equal distributions of sex/gender, as more than 90% of participants in the present study reported being female. Future research could also include objective assessments of physical activity, such as via accelerometry, and longer-term follow-up assessments. One positive feature of studying an existing and ongoing series of hiking challenges is that the intervention has built-in potential for sustainability, with the potential for longer-term effects heightened among those intervention-group participants who elected to continue their engagement by signing up for the next (i.e., Summer) hiking challenge on their own.

Overall, the present studies demonstrate potential of continued research in this area. Efforts to address additional reported barriers can elucidate whether these efforts further increase uptake of this intervention and augment health and well-being benefits. The current findings support the potential of such efforts, with statistically significant intervention effects on hiking frequency in Study 2 and corresponding evidence of positive impacts on aspects of well-being, including sleep duration and quality. Future research examining the feasibility and acceptability of this intervention approach and its effects on daily activities and various aspects of health and well-being in a larger, more diverse sample is warranted, as this research has the potential to promote physical activity and well-being at times when physical activity rates are low.

## Electronic supplementary material

Below is the link to the electronic supplementary material.

Supplementary Material 1

Supplementary Material 2

## Data Availability

The datasets from this study are available from the corresponding author on reasonable request.
