# Supplementary material for: Effects of a randomized controlled hiking intervention on daily activities, sleep, and stress among adults during the COVID-19 pandemic
Source: BMC Public Health. 2023 May 15;23:892. doi: 10.1186/s12889-023-15696-7 (PMC10184062; doi:10.1186/s12889-023-15696-7)
Supplement: Supplementary file 1 — Supplementary Material 1 [file 12889_2023_15696_MOESM1_ESM.docx]

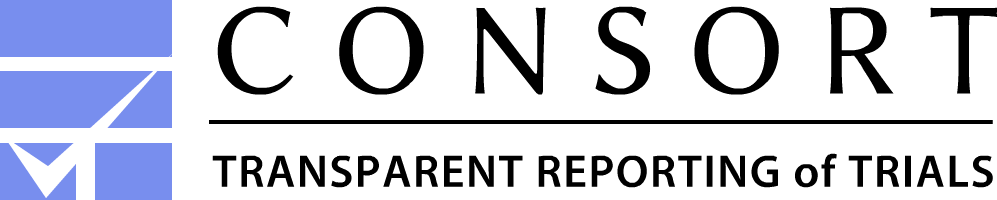


**Electronic Supplementary Materials 1: Study 1 Flow Diagram**

## Follow-Up

Analysed (n=22)
♦ Excluded from analysis (n=0)

## Analysis

Analysed (n=19)
♦ Excluded from analysis (n=0)

Lost to follow-up (n=1; medical condition prevented participation; (n=6 did not engage beyond baseline, unknown reasons)

Lost to follow-up (n=5 did not engage beyond baseline; unknown reasons)

## Enrollment

Allocated to control (n=27)

♦ Provided allocated intervention (n=27)

♦ Did not receive allocated intervention (n=0)

## Allocation

Allocated to intervention (n=26)

♦ Provided allocated intervention (n=26)

♦ Did not receive allocated intervention (n=0)

Randomized (n=53)

Excluded (n=293)

♦  Not meeting inclusion criteria (n=161)

♦  Declined to participate (n=83)

♦  Other reasons (n=48; completed the screener and/or consent after the study had already been filled; n=1; failed to complete baseline procedures)

Assessed for eligibility (n=346)
