# Supplementary material for: Effects of a randomized controlled hiking intervention on daily activities, sleep, and stress among adults during the COVID-19 pandemic
Source: BMC Public Health. 2023 May 15;23:892. doi: 10.1186/s12889-023-15696-7 (PMC10184062; doi:10.1186/s12889-023-15696-7)
Supplement: Supplementary file 2 — Supplementary Material 2 [file 12889_2023_15696_MOESM2_ESM.docx]

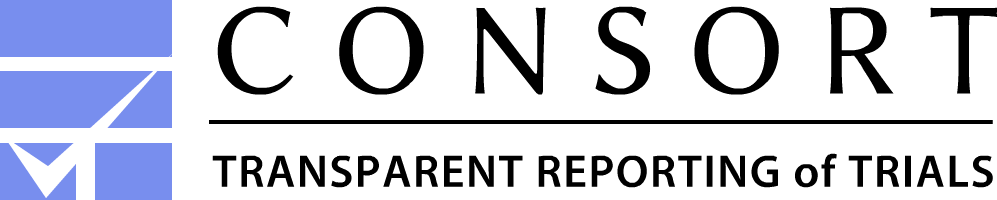


**Electronic Supplementary Materials 2: Study 2 Flow Diagram**

Excluded (n=156)

♦  Not meeting inclusion criteria (n=45)

♦  Declined to participate (n=70)

♦  Other reasons (n=39; completed the screener and/or consent after the study had already been filled; n=2; failed to complete baseline procedures)

## Follow-Up

Analysed (n=25)
♦ Excluded from analysis (n=0)

## Analysis

Analysed (n=22)
♦ Excluded from analysis (n=0)

Lost to follow-up (n=3; did not engage beyond baseline, reason unknown)

Lost to follow-up (n=1; did not engage beyond baseline, reason unknown)

## Enrollment

Allocated to control (n=26)

♦ Provided allocated intervention (n=26)

♦ Did not receive allocated intervention (n=0)

## Allocation

Allocated to intervention (n=25)

♦ Provided allocated intervention (n=25)

♦ Did not receive allocated intervention (n=0)

Randomized (n=51)

Assessed for eligibility (n=207)
